# Gelam honey potentiates ex vivo corneal keratocytes proliferation with desirable phenotype expression

**DOI:** 10.1186/s12906-016-1055-7

**Published:** 2016-02-24

**Authors:** Alia Md Yusof, Norzana Abd Ghafar, Taty Anna Kamarudin, Chua Kien Hui, Yasmin Anum Mohd Yusof

**Affiliations:** Department of Anatomy, Universiti Kebangsaan Malaysia Medical Centre (UKMMC), Jalan Yaacob Latif, Bandar Tun Razak, Cheras, 56000 Kuala Lumpur, Malaysia; Department of Physiology, Universiti Kebangsaan Malaysia Medical Centre (UKMMC), Jalan Yaacob Latif, Bandar Tun Razak, Cheras, 56000 Kuala Lumpur, Malaysia; Department of Biochemistry, Universiti Kebangsaan Malaysia Medical Centre (UKMMC), Jalan Yaacob Latif, Bandar Tun Razak, Cheras, 56000 Kuala Lumpur, Malaysia

**Keywords:** Gelam honey, Corneal keratocytes, Proliferation, Phenotypes

## Abstract

**Background:**

This study aimed to evaluate the effects of Gelam honey on corneal keratocytes proliferative capacity and phenotypic characterization via MTT assay, gene expression and immunocytochemistry.

**Methods:**

Corneal keratocytes from New Zealand white rabbits were cultured in basal medium (BM) and serum enriched medium (BMS). Serial dilutions of Gelam honey (GH) were added to both media and cells were cultured until passage 1. MTT assay was performed on corneal keratocytes in both media to ascertain the optimal dose of GH that produced maximum proliferation.

**Results:**

Gelam honey at the concentration of 0.0015 % in both media showed the highest proliferative capacity with no morphological changes compared to their respective controls. The gene expression of aldehyde dehydrogenase (ALDH), a marker for quiescent keratocytes and vimentin, a marker for fibroblast, were higher in the GH enriched groups. The alpha smooth muscle actin (α-SMA) expression, marker for myofibroblast, was lower in GH treated groups compared to the controls. Immunocytochemistry results were in accordance to the gene expression analyses.

**Conclusion:**

Gelam honey at a concentration of 0.0015 % promotes ex vivo corneal keratocytes proliferation while retaining desirable phenotype expression. The results serve as a basis for the development of Gelam honey as a potential natural product in promoting corneal wound healing.

## Background

Cell proliferation is the key process in wound healing and it involves several metabolic processes which require much energy. Glucose is the main source of energy for metabolism of most cells, either in vivo or ex vivo.

Honey is one of the natural products that is rich in glucose and contains enzymes, amino acids, vitamins and minerals [1]. It possesses medicinal properties which include antimicrobial, antioxidant, antimutagenic, antitumour, anti-inflammatory and the capability to stimulate cell proliferation [2]. Honey proliferative effects on the in vivo study of the skin wound healing were reported earlier [3–6]. However, to date, there is lacunae in the existing scientific literature with regard to the proliferative effects of honey on in vitro corneal cells, particularly concerning Gelam honey (GH).

GH is one of the Malaysian monofloral honeys that is found in bee hives of Gelam tree. It contains the highest total phenols and flavonoid content [7]. The total sugar content of GH was reported to be the highest among all Malaysian monofloral honeys [8]. Previous research studies revealed that GH possessed the capability in accelerating skin wound healing [9–12]. In addition, GH has been reported to increase the tensile strength of wounded dermal layer upon healing which is important in ensuring a strong protective dermis [13]. GH is also reported to possess antibacterial effects [14], capable of minimising oedema and pain sensation during inflammation [15]. It is also reported to possess high antioxidant content [16]. To the best of our knowledge, there are no reported studies of GH on corneal injury. Since, the skin cells and the corneal cells are of epithelial in nature [17], it generates much interest to study the effects of GH on corneal cells.

The present study used primary culture of rabbit corneal keratocytes to evaluate the proliferative effects of GH at the cellular level, via MTT assay, morphological evaluation, gene and protein expression analyses. The results of the present study exhibited the positive role of GH in promoting the proliferation and expression of desirable ex vivo corneal keratocytes phenotypes. These findings may be beneficial in the treatment of corneal stroma wound healing.

## Methods

This research was approved by the Universiti Kebangsaan Malaysia Research and Animal Ethics Committee (PP/ANAT/2011/NORZANA/21-JULY/378-AUGUST-2011-JULY2012-AR-CAT2).

### GH preparation

GH was obtained from the Apiary unit, Department of Agriculture, Malaysia. The honey was aliquoted into several air tight glass bottle containers and gamma irradiated at 25 kGy [18] at the Malaysian Nuclear Agency. The honey was then kept at room temperature.

### Corneal keratocytes isolation and cell culture

Corneas from six New Zealand white strain rabbits (*n* = 6) were obtained from a local slaughterhouse and processed within 2 hours of post slaughtering [19]. The corneas were extracted and ocular muscles, iris and endothelium were removed. The loosened epithelial layer was scraped off after immersing the corneas for approximately 18 h in Dispase 2 mg/ml (Gibco Invitrogen, USA). Then, the stromal tissues were cut into eight pieces and digested with 0.3 % Collagenase type I (Gibco Invitrogen, USA) for about 2 hours at 37 °C in a constant shaker at 200 rpm. Digested stroma and the isolated keratocytes were centrifuged for 10 min at 500 g and the resultant cell pellet was washed thoroughly with phosphate buffered solution (PBS pH 7.2, Gibco Invitrogen, USA).

Total cell quantification of corneal keratocytes was done using Trypan blue dye (Gibco Invitrogen, USA) and haemocytometer (Weber Scientific Int, England). The keratocytes were then seeded at a density of 1 × 10^5^ cells/cm^2^ in six well plates (BD Falcon, NJ) in basal medium with serum (BMS), which consist of F12: DMEM (1:1 ratio) (Gibco Invitrogen, USA), 10 % foetal bovine serum (FBS, Gibco Invitrogen, USA) and 1 % of antibiotic-antimycotic (Gibco Invitrogen, USA), vitamin C (Gibco Invitrogen, USA) and Glumatax (Gibco Invitrogen, USA).

Cultures were kept in the incubator (Jouan Duguay Troin, SH) at 37 °C, 5 % CO_2_ and 95 % humidity until confluent. The medium was changed every 2 days. The morphological changes and cell growth were observed daily to detect signs of contamination. Upon confluence, the cells were trypsinized with warm 0.125 % trypsin EDTA (Gibco Invitrogen, USA). The cells were then subcultured to passage 1.

### MTT 3-[4, 5-dimethyl thiazolyl-2]-2, 5-diphenyl tetrazolium bromide assay

MTT assay (Sigma-Aldrich) was used for quantitative analysis of GH on corneal keratocytes proliferation and viability. Corneal keratocytes from passage 1 were cultured in BMS medium at the density of 5 × 10^3^ cells/cm^2^ in a 96 well plate (Cellstar Greiner Bio-one, Germany). After 24 h, the medium was replaced with basal medium (BM) and new BMS medium supplemented with different concentrations of GH in serial dilution from 0 to 1.56 %. The cells were incubated at 37 °C, 5 % CO2 and 95 % humidity for 48 h.

In brief, 10 μl of MTT solution was added into each well and incubated for 4 h. MTT solution was then removed and solubilized with 200 μl of dimethylsulfoxide (DMSO). Absorbance was measured at 570 nm by Versamax Microplate Reader. GH which gave the highest cell proliferation was chosen as the optimal concentration for further tests involving protein and gene expression analyses. The optimal dose of GH was identified at 0.0015 % concentration.

### Gene expression analyses

Total RNA extraction was done using the TRIzol RNA isolation method, based on manufacturer’s protocol. An amount of 1 ml of TRI reagent solution (Molecular Research Centre, USA) was added to all culture dishes at Day 3 to lyse the cells. Then, 200 μl chloroform was added to form phase separation. After that, 500 μl isopropanol and 5 μl polyacryl carrier (Molecular Research Centre, USA) were added to precipitate total RNA. The RNA pellet was then washed with 75 % ethanol and air dried, before adding 20 μl Rnase and Dnase free distilled water (Invitrogen, USA) to dissolve the RNA.

Complimentary DNA (cDNA) synthesis was carried out using Enzynomics TOPscript™ cDNA Synthesis Kit, according to manufacturer’s protocol. In brief, primer annealing was done for 10 min at 23 °C, reverse transcription for 60 min at 50 °C and reaction termination for 5 min at 85 °C.

The qRT-PCR was performed on three marker genes related to corneal keratocytes and its phenotypes i.e. Aldehyde dehydrogenase (ALDH) as a marker for quiescent keratocytes, Vimentin as a marker for activated fibroblast and Alpha smooth muscle actin (α-SMA) as a marker for myofibroblast phenotype. Glyceraldehyde 3-phosphate dehydrogenase (GAPDH) was used as the house keeping gene. The primers were designed from NIH Genbank and tabulated (Table 1).

**Table 1:** Description of primers used in qRT-PCR

Gene	Sequence 5’ → 3’	NIH Genbank access no.	PCR product size (bp)
GAPDH	F: caacgaatttggctacagca	NM_001082253	186
	R: aaactgtgaagaggggcaga		
ALDH	F: gagtggcatgattcagtgagc	AY503694	186
	R: gagtagtcgtcccctcttgga		
Vimentin	F: tgcaggaagagattgccttt	AY465353	117
	R: tgaggtcaggcttggagaca		
α-SMA	F: tcgacatcaggaaggacctct	X60732	206
	R: catctgctgaaaggtggacag		

Two steps qRT-PCR was done with GENET BIO 2X Prime Q-Master Mix (SYBR Green I) on Bio-Rad icycler machine (Bio-Rad, USA). The reaction cycle conditions were, cycle 1: 95 °C for 3 min (1x), cycle 2: 94 °C for 10 s, followed by 61 °C for 30 s (40x), followed by melting curve analysis.

The specificity and the PCR product size were confirmed by 2 % gel electrophoresis (Invitrogen, USA) and was photographed by Polaroid MP4 camera (Polaroid DM Scientific).

### Immunocytochemistry

Immunocytochemistry staining was carried out using the Abcam Dako Immunostaining Kit protocol with some modifications. The culture slides in each group were fixed with 4 % paraformaldehyde at Day 3 (day of confluence). The slides were treated with acetone at 4 °C for 5 min and followed by incubation with 0.03 % hydrogen peroxide for 5 min at room temperature. The slides were then heated for 20 min at 95 °C, cooled with running water and soaked with primary antibodies for 30 min.

Antibodies such as ALDH, Vimentin and α-SMA antibodies (Abcam) with the dilution of 1:200 for ALDH and α-SMA and 1:400 for Vimentin. Slides were then incubated with secondary antibody for 30 min, followed by DAB substrate for 10 min and counterstained with haematoxylin (Sigma-Aldrich). The slides were examined under a light microscope (Leica) with Image Analyser 5.2 Morphology Software.

### Data analysis

All data were analysed using Statistical Package for Social Sciences (SPSS) version 21. Values were expressed as mean ± standard error of mean (SEM) using Student’s *t*-test. A *p* value ≤ 0.05 was considered to be significant.

## Results

### MTT assay

**Fig. 1:**
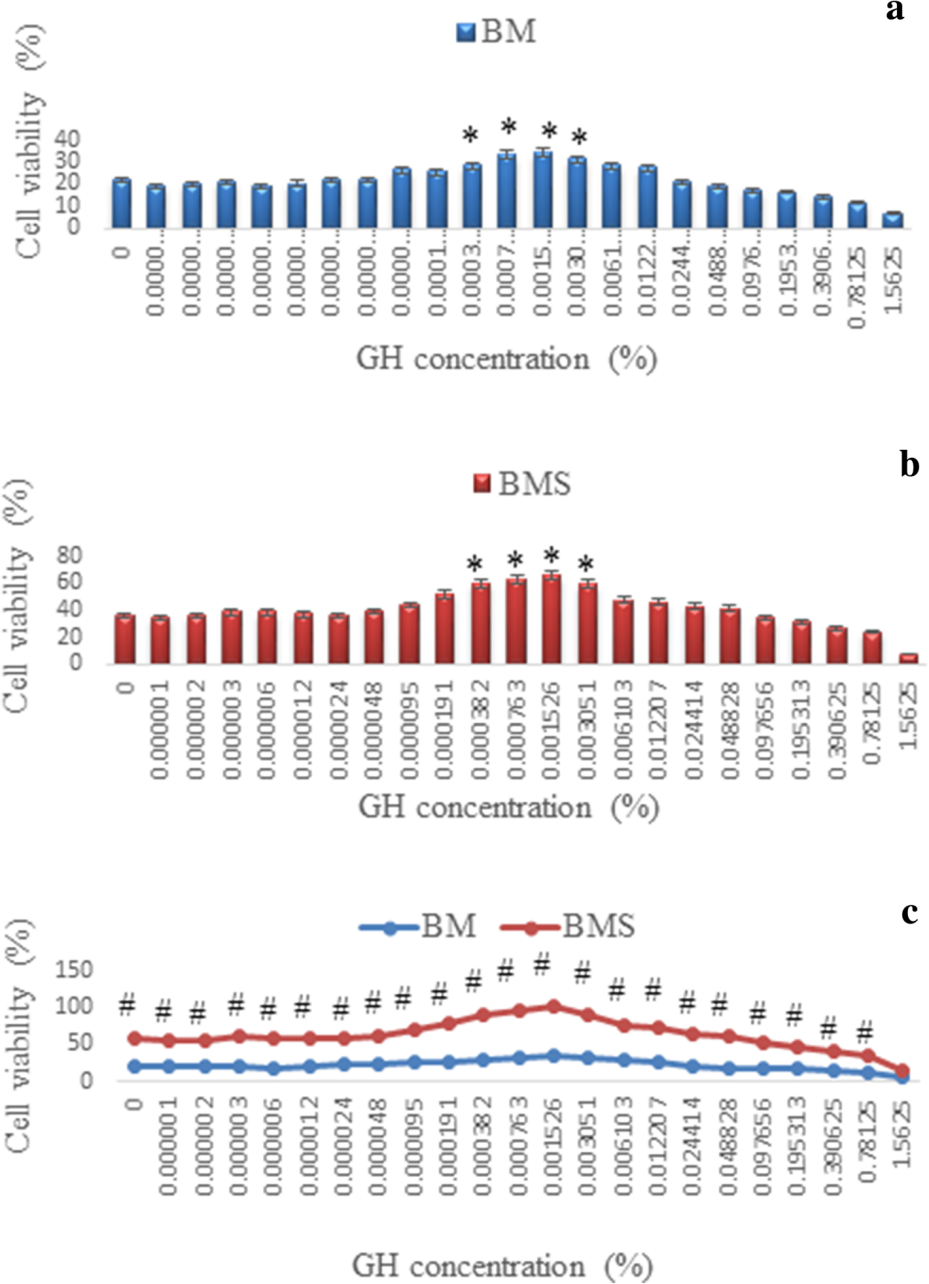
Corneal keratocytes proliferation in (**a**) Basal medium (BM), (**b**) Basal medium with serum (BMS), supplemented with serial dilution of GH and (**c**) comparison between the two media, BM and BMS. * denotes significant difference compare to control in the same group (*p* < 0.05), # denotes significant difference at the same concentration in different groups (*p* < 0.05)

Figure 1 showed the MTT assay of corneal keratocytes cultured in serial dilutions of GH. Corneal keratocytes showed higher proliferation in the BMS group compared to the BM group in all GH concentrations (*p* < 0.05). GH at the concentration of 0.0004 to 0.0031 % in both media demonstrated a significant increase in keratocytes proliferation compared to media devoid of GH (*p* < 0.05). Keratocytes cultured in BMS medium supplemented with 0.0004 to 0.0031 % GH significantly increased keratocytes proliferation compared to BM supplemented GH medium. There was no significant difference in cell proliferation between 0.0004 and 0.0031 % GH in both media. Therefore, GH at the concentration of 0.0015 % was chosen as the optimal dose.

### Cell morphology

**Fig. 2:**
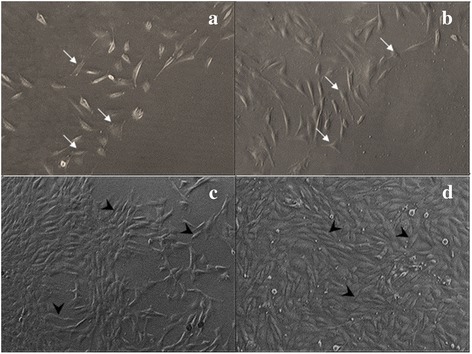
Phase contrast micrographs of corneal keratocytes cultured in four different media. **a** Basal medium (BM), (**b**) BM + 0.0015 % GH, (**c**) Basal medium with serum (BMS) and (**d**) BMS + 0.0015 % GH. Micrographs were taken at Passage 1 Day 3 of culture. Cell density was the least in BM medium (**a**) and the highest in BMS + 0.0015 % GH (**d**). Corneal keratocytes cultured in BM only (**a**) and with GH (**b**) exhibited shorter and broader cell morphology (*thin arrow*) compared to that of elongated and fibroblastic-shaped cells (*thick arrow*) in BMS medium (**c**) and with GH (**d**). Magnification (x50)

Phase contrast micrographs of corneal keratocytes in four different culture media with or without 0.0015 % GH at Day 3 were shown in Fig. 2. The morphology of corneal keratocytes cultured in BM and BM + 0.0015 % GH (Fig. 2a and b) demonstrated shorter and broader cell with several cytoplasmic processes resembling dendritic cells. In contrast, corneal keratocytes cultured in BMS with or without GH (Fig. 2c and d) exhibited elongated, spindle-shaped cell resembling fibroblasts. Cell density in BM (Fig. 2a) was lesser than BMS (Fig. 2c). The addition of 0.0015 % GH in both media resulted in higher cell density than their respective controls. Keratocytes in BMS with 0.0015 % GH showed the highest cell density and attained confluence (Fig. 2d).

### Gene expression analyses

**Fig. 3:**
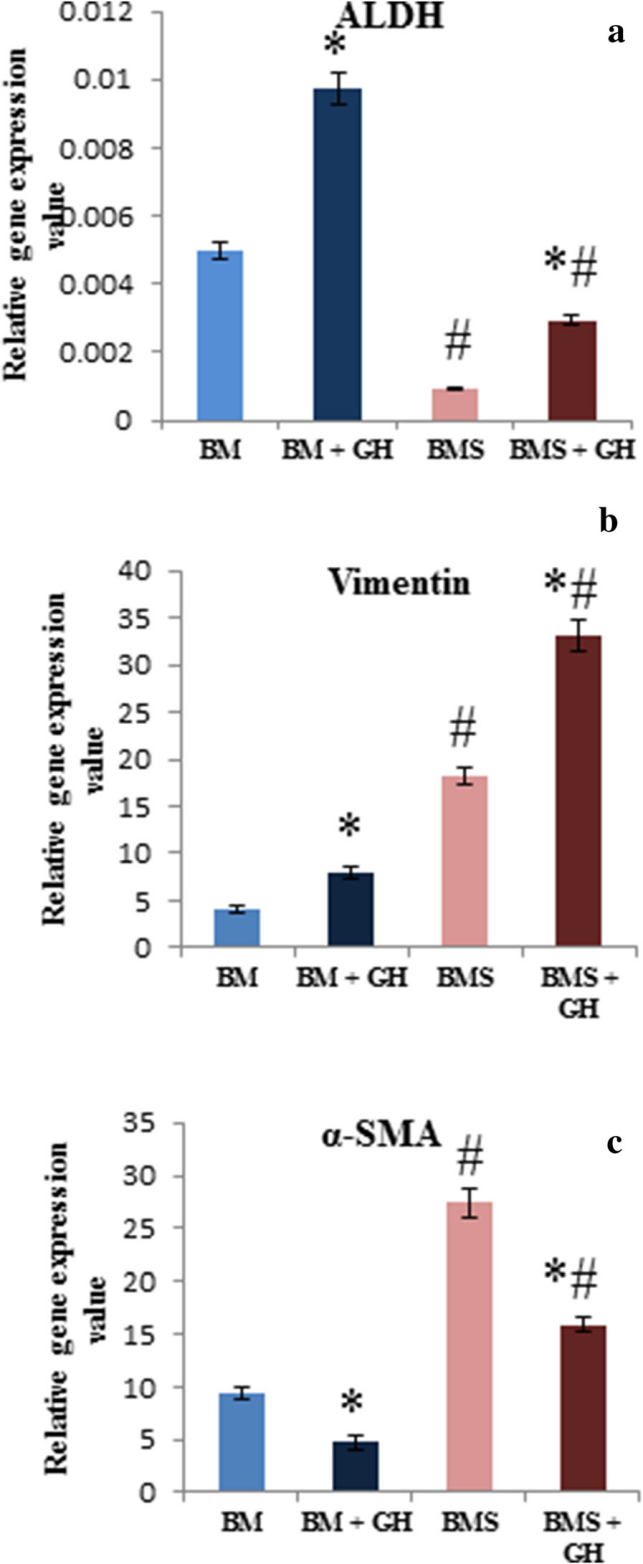
Gene expression of (**a**) ALDH, (**b**) Vimentin and **c** α-SMA of corneal keratocytes cultured in BM, BM + GH, BMS and BMS + GH. * denotes significant difference in the same group (*p* < 0.05), # denotes significant difference between different groups (*p* < 0.05)

The expression of ALDH was higher in BM compared to BMS with or without GH supplementation (Fig. 3b). However, with the addition of 0.0015 % GH to both media, the expression of ALDH gene increased significantly (*p* < 0.05) compared to their respective controls. Expression of ALDH was the highest in the BM group supplemented with 0.0015 % GH.

There was a significant increase in the Vimentin expression of corneal keratocytes cultured in both BM and BMS with 0.0015 % GH compared to their respective controls (Fig. 3c). Keratocytes cultured in BMS supplemented with GH showed the highest Vimentin expression compared to the other groups.

Expression of α–SMA gene was higher in the BMS groups compared to the BM groups (Fig. 3d). The addition of 0.0015 % GH in both media demonstrated significantly lower expression of α–SMA compared to their respective controls (*p* < 0.05). The gene expression of α–SMA was the lowest in the BM supplemented with 0.0015 % GH.

**Fig. 4:**
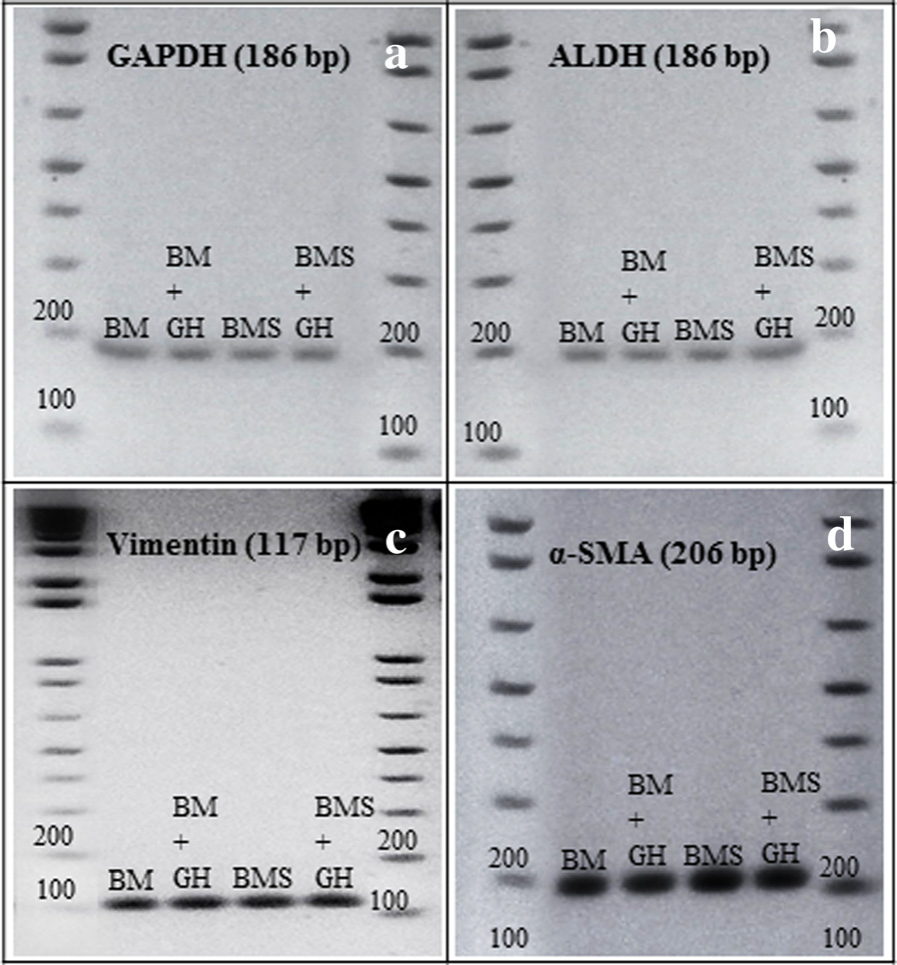
Gene expression of (**a**) GAPDH, **b** ALDH, **c** Vimentin and **d** α-SMA of corneal keratocytes cultured in BM, BM + GH, BMS and BMS + GH. * denotes significant difference in the same group (*p* < 0.05), # denotes significant difference between different groups (*p* < 0.05)

Gel electrophoresis of ALDH, Vimentin and α–SMA genes demonstrated the specificity and stability of corneal keratocytes phenotypes in all culture groups (Fig. 4).

### Immunocytochemistry (ICC)

**Fig. 5:**
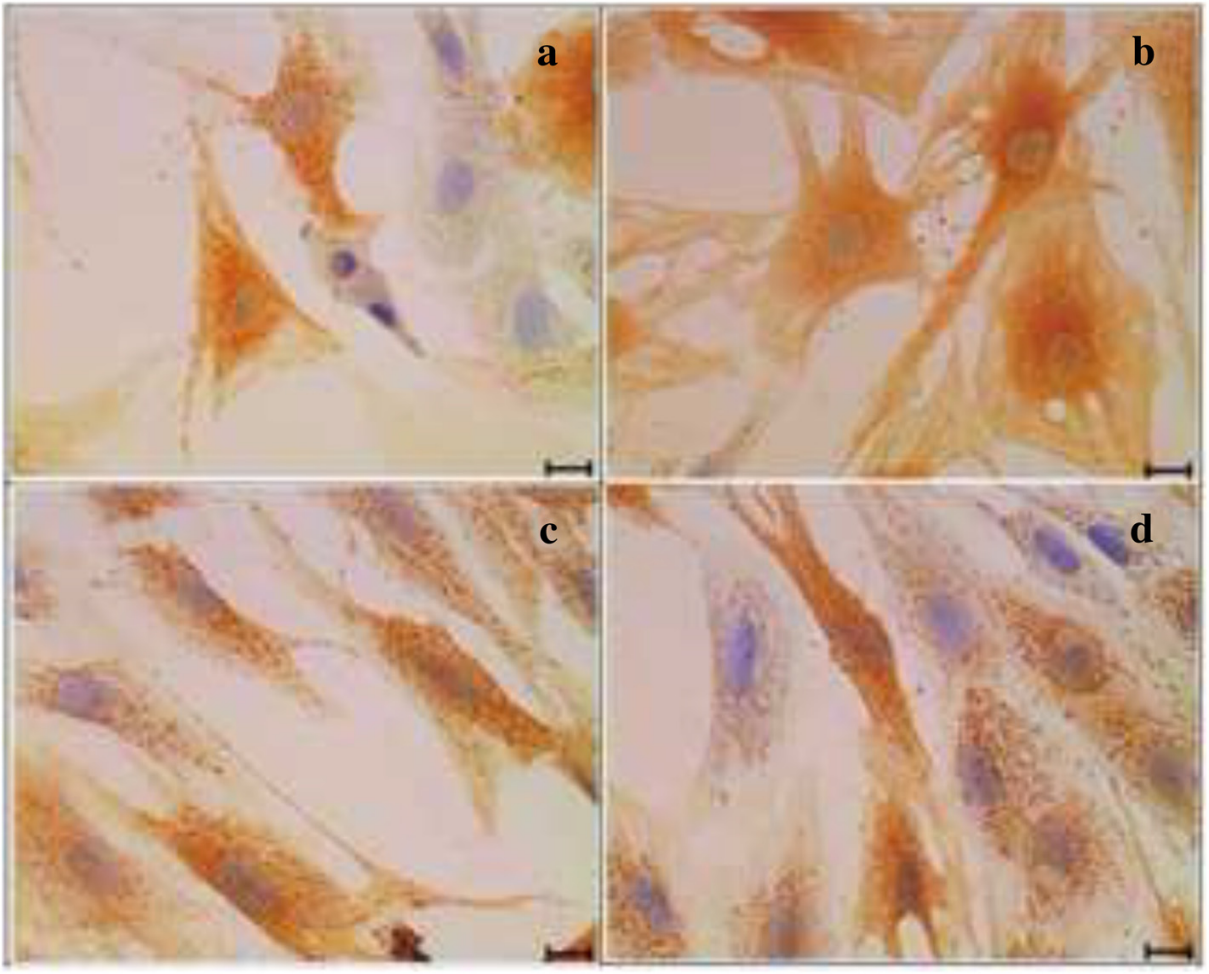
Immunocytochemistry of ALDH in four different media. **a** Basal medium (BM), (**b**) BM + 0.0015 % GH, (**c**) Basal medium with serum (BMS) and (**d**) BMS + 0.0015 % GH. Corneal keratocytes expressed stronger intensity of ALDH antibody in basal medium (**a**) compared to serum enriched basal medium (**c**) and also in the GH enriched basal medium (**b**) as compared to serum enriched medium with GH (**d**). Scale bar represents 20 μm

The ICC slides were examined qualitatively according to the presence of positive stained cells. This qualitative procedure was based on previous research studies [20, 21]. The intensity of the ALDH protein stain was stronger in the BM group (Fig. 5a) compared to the BMS group (Fig. 5c). The addition of 0.0015 % GH in the BM group (Fig. 5b) exhibited stronger intensity of this protein compared to that of GH supplemented BMS group (Fig. 5d).

**Fig. 6:**
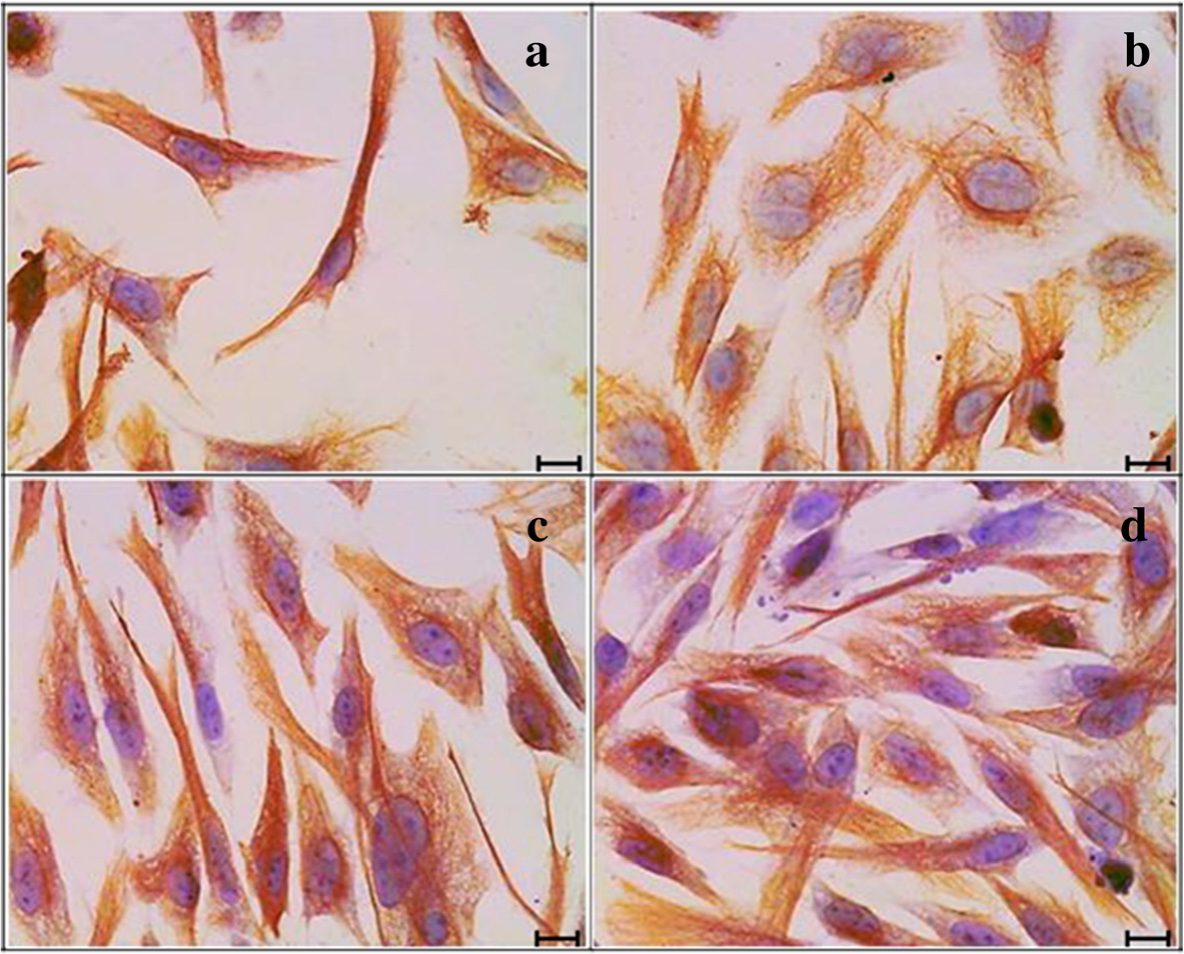
Immunocytochemistry of Vimentin in four different media. **a** Basal medium (BM), (**b**) BM + 0.0015 % GH, (**c**) Basal medium with serum (BMS) and (**d**) BMS + 0.0015 % GH. Vimentin protein was expressed in all culture groups. The strongest intensity was observed in the serum enriched medium with GH (**d**). Scale bar represents 20 μm

Corneal keratocytes showed positive stained cells for Vimentin in all culture groups (Fig. 6a-d). However, corneal keratocytes in BMS supplemented with 0.0015 % GH exhibited the strongest intensity of positive stained Vimentin protein (Fig. 6d).

**Fig. 7:**
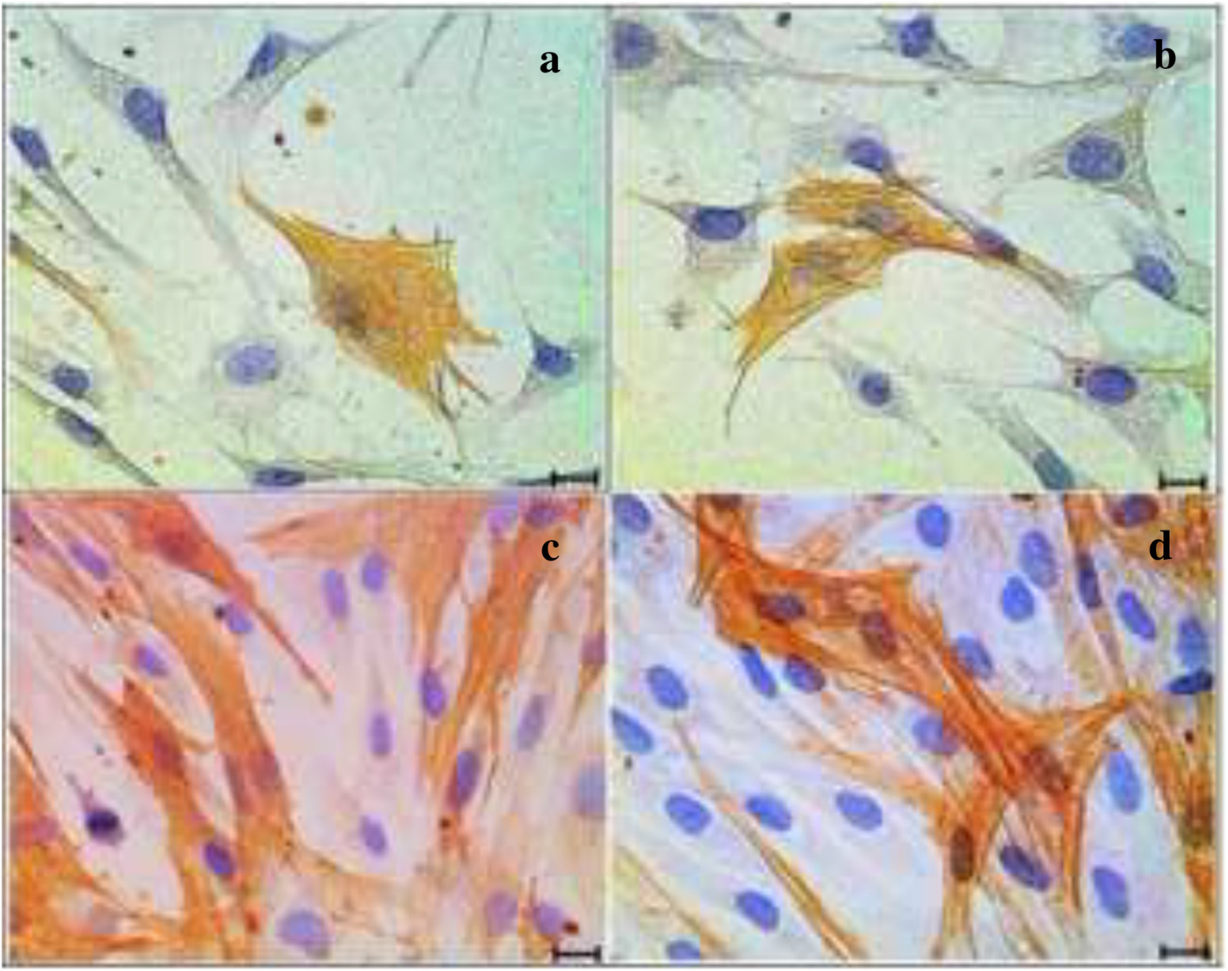
Immunocytochemistry of α-SMA in four different media. **a** Basal medium (BM), (**b**) BM + 0.0015 % GH, (**c**) Basal medium with serum (BMS) and (**d**) BMS + 0.0015 % GH. Positive stained cells showed stronger intensity in serum enriched media (**c**) and (**d**) compared to basal media (**a**) and (**b**). Scale bar represents 20 μm

The intensity of the protein expression of α–SMA was stronger in the BMS groups (Fig. 7c, d) compared to the BM groups (Fig. 7a, b). The addition of 0.0015 % GH to BM (Fig. 7b) exhibited weaker expression compared to that of GH added BMS group (Fig. 7d).

## Discussion

Cell proliferation is vital for wound healing and it requires a considerable amount of glucose as the main source of energy. Ex vivo cells obtain glucose from the culture medium [22] and serum [23] for metabolic process, growth and proliferation. Glucose is processed in cell mitochondria to produce adenosine triphosphate (ATP) [24], a form of energy which is crucially needed for cellular metabolism including cell mitoses or proliferation [25]. In the present study, the glucose content in 0.0015 % GH may have increased glycolytic enzymes such as glyceryldehyde-3-phosphate dehydrogenase, lactate dehydrogenase and glucose-6-phosphate dehydrogenase. These enzymes are clues to on-going cellular energy production [26]. GH may have induced protein and DNA synthesis during the S phase of cell cycle, thus enhancing cell mitoses and proliferation. The present study demonstrated the addition of GH at the concentration of 0.0015 % showed a positive role in ex vivo corneal keratocytes proliferation. These findings are in accordance to a previous report on Tualang honey in serum enriched media which demonstrated positive proliferative effects on osteoblast cell line [27].

The present study also showed the corneal keratocytes morphology cultured in GH enriched media was comparable to controls. It was reported dendritic-shaped corneal keratocytes cultured in serum free media [28] transformed to fibroblastic-shaped when cultured in serum enriched media [29]. The present study has proven that GH maintained corneal keratocytes morphology either in serum free or serum enriched media. These findings indicate that GH did not contain any elements that could cause unfavourable morphology transformation of corneal keratocytes. This attribute is important for its application in ex vivo cell study or in cell-based therapy.

ALDH is a corneal crystalline that functions as a UV protector, antioxidant and maintaining corneal transparency [30]. It is a molecular marker for quiescent keratocytes [31]. The production of ALDH has been reported to be located in the keratocytes nuclei and cytoplasm [32]. As keratocytes transformed to fibroblast and myofibroblast during wound healing or due to the presence of serum or growth factors in ex vivo culture medium, the ALDH protein was no longer expressed [33]. The present study demonstrated the capability of GH to preserve more corneal crystalline which was evidenced by higher gene expression of ALDH. These findings signified the potentiality of GH to be used in the treatment of eye disorders, since it retains corneal transparency. These findings are vital in the incorporation of ex vivo corneal keratocytes in cell-based therapy for maintaining normal clarity and function of the cornea.

Another crucial corneal keratocytes phenotype marker is Vimentin which is an active keratocytes phenotypes, fibroblasts, and represents a major intracellular cytoskeletal protein of fibroblast [34]. Absence of Vimentin in fibroblast resulted in weak intra skeletal structure, low fibroblast mechanical stability and diminished contractility [35], thus delaying the process of wound healing. Profusion of fibroblast was also an advantage during corneal wound healing since it promoted extracellular matrix productions during wound closure [36]. The results of the present study revealed high expression of Vimentin in GH added media compared to controls with reciprocal reduction of ALDH expression in the same media. Hence, GH may have the potential to promote ex vivo corneal wound healing by increasing transformation of more quiescent keratocytes into active fibroblasts. Since fibroblast transformation is induced in the presence of platelet derived growth factor (PDGF) and fibroblast growth factor (FGF) [37], we hypothesise that molecular components in GH may act in a similar pathway as PDGF and FGF in inducing fibroblast transformation.

The α–SMA is an intracellular actin isoform in myofibroblast that involves in cell contractility, structure and integrity [38]. Its high expression is usually closely related to scar formation [39]. In a standard corneal keratocytes culture, i.e. in BMS, the α–SMA expression was usually high compared to the media without serum due to the presence of transforming growth factor β (TGF-β) [40]. However, in the present study, the addition of GH in BMS significantly reduced the expression of α–SMA with a concurrent high expression of Vimentin. This indicates the possibility of reversal transformation of myofibroblast to fibroblast in GH supplemented media. Reversal transformation is possible in the presence of FGF-heparin [41]. There is a possibility of GH possessing the same growth factor or any molecular component that may function in a similar way to FGF-heparin by promoting the reverse transformation. The increased amount of active repair phenotype, fibroblast and the decreased number of scar phenotype, myofibroblast, may result in an accelerated corneal wound healing process with less risk of developing corneal scar formation. To date, there is a paucity of studies with regard to topical use of honey-based eye drop in the treatment of corneal ulcer or corneal cell-based therapy. Hence, the results of the present study assume much importance with regard to GH being used as a supplementary treatment for this ailment.

The immunocytochemistry findings on ALDH, Vimentin and α–SMA protein were in accordance with the respective gene analyses findings.

## Conclusion

GH at the concentration of 0.0015 % stimulates corneal keratocytes ex vivo proliferation while preserving corneal crystalline, transforming more activated keratocytes; fibroblast and producing less myofibroblast. These encouraging characteristics of corneal keratocytes cultured in GH are fundamental findings that may open the door for future studies in realizing GH as a potential nutripharmaceutical agent to be administered in the form of topical eye drop for the treatment of corneal wound healing. In addition, the positive GH proliferative effects may initiate a new paradigm in using GH as an effective supplement into the standard corneal keratocytes culture medium for corneal keratocytes expansion, corneal cell transplantation or in the development of bioengineered cornea. Further studies on exploration of GH components are needed in order to ascertain the favourable effects on corneal keratocytes proliferation and desirable phenotypes expression.
